# Interest of a standardized hypnotic message for the reduction of pain and anxiety in cancer patients treated by capsaicin patch for neuropathic pain: a randomized controlled trial

**DOI:** 10.1186/s12906-021-03329-8

**Published:** 2021-05-27

**Authors:** Rémi Etienne, Myriam Laurent, Aline Henry, Antoine Bioy, Julia Salleron, Cécile Huin Schohn, Nathalie Cretineau

**Affiliations:** 1 Department of Supportive Care in Oncology, Institut de Cancérologie de Lorraine, Université de Lorraine, F-54519 Vandœuvre-lès-Nancy, France; 2 University of Paris 8, Laboratory of Psychopathology and Neuropsychology, St Denis, Paris, France; 3 Departement of biostatistics, Institut de Cancérologie de Lorraine, Université de Lorraine, F-54519 Vandœuvre-lès-Nancy, France; 4 Research Department, Institut de Cancérologie de Lorraine, Université de Lorraine, F-54519 Vandœuvre-lès-Nancy, France

**Keywords:** Cancer, Pain, Anxiety, Hypnosis, Music therapy

## Abstract

**Background:**

Neuropathic pain is characterized by spontaneous painful symptoms. Medical therapies include the use of a capsaicin 8% patch (Qutenza®, Grünenthal Gmbh, Germany), and patients may experience a sharp burning sensation at application and removal of the patch. This study aimed to evaluate the impact of playing a standardized hypnosis recording during application, on the pain and anxiety induced by capsaicin treatment.

**Methods:**

In a randomized, controlled trial, we assessed the benefits of the intervention firstly on pain and secondly on anxiety, as measured using numerical rating scales. All patients had application of the capsaicin patch, including the possibility for the patient to apply a cold patch. Participants were randomly assigned to one of 3 groups, namely the “Standard group” (no intervention), “Hypnosis group”, in which a standardized hypnotic message was played during application, or the “Music group” in which relaxing music was played during application of the patch.

**Results:**

Sixty-nine patients were included. Overall, there was no significant difference in pain scores between groups (*p* = 0.355). Compared to standard application, anxiety was significantly lower in the hypnosis group after application (*p* = 0.007), with no significant difference between the standard and music arms (*p* = 0.271), or between the hypnosis and music arms (*p* = 0.423).

**Conclusions:**

Listening to a standardized hypnotic message during application of a capsaicin patch was found to significantly lower anxiety. These findings indicate that the use of a hypnotic message can reduce discomfort and warrant its evaluation in other indications of pain or anxiety during treatment procedures.

**Trial registration:**

NCT02822625.

## Background

The International Association for the Study of Pain (IASP) defines neuropathic pain (NP) as “pain that arises as a direct consequence of a lesion or disease affecting the somatosensory system”. The world prevalence of NP in the general population varies from 6.9 to 10% [1]. NP can lead to a significant decline in quality of life, and represents a major public health challenge. In 60% of cases, NP is localized within a specific area of the body [2]. NP is characterized by specific symptoms that can associate sensations that are not specifically painful, but rather unpleasant (numbness, tingling, itching), with spontaneous painful symptoms (stabbing pain, feeling of pressure, burning or electric shock sensation, but also allodynia or hyperalgesia), as well as negative sensory signs (impaired perception of mechanical or thermal stimuli) [1].

Improvements in our understanding of the pathophysiology of NP have led to the emergence of medical and non-medical therapies, which are generally proposed in a pluridisciplinary context. Medical therapies include the use of local topical applications such as 5% lidocaine, plasters or an 8% capsaicin patch (Qutenza®, Grünenthal Gmbh, Germany) [3]. Capsaicin is a highly selective agonist of transient receptor potential vanilloid 1 (TRPV1), which is present in the skin and essential for pain signalling [4]. The capsaicin patch is indicated in association with other antalgic agents in the management of peripheral NP that is localized, assessable and restricted to a limited area, in non-diabetic adults.

Despite the agonistic activity, exposure to capsaicin applied topically at high doses leads to initial activation of nociceptive nerve fibers expressing TRPV1, causing a burning sensation and erythema. Then, after exposure to capsaicin, TRPV1-containing sensory axons are desensitized, with down-regulation of TRPV1 expression. Consequently, cutaneous nociceptors become less sensitive, leading to pain relief. The changes induced in cutaneous nociceptors by capsaicin are reversible and their normal function can be restored within a few weeks [5]. The most commonly described adverse effects (AEs) include a sharp burning sensation on application and removal of the patch, as well as erythema and itching [6, 7]. Although these AEs are transient and totally reversible, they may increase discomfort, leading to premature removal of the patch in 2 to 6% of patients [8].

The difficulty of controlling these AEs prompted us to envisage the use of therapeutic hypnosis as a complementary strategy on top of standard treatment, which generally associates the use of painkillers with application of cold. According to the American Psychological Association, therapeutic hypnosis can be defined as a state of consciousness involving focused attention and reduced peripheral awareness characterized by an enhanced capacity for response to suggestion. Since the end of the 1990s, the field of neurosciences has confirmed the existence of a unique neurological functioning occurring during hypnosis, notably by demonstrating how suggestions of pain relief can modify the sensory and affective components of the pain process [9]. Numerous studies have now demonstrated the positive impact of therapeutic hypnosis for pain management [10], in particular to reduce pain and anxiety in patients with burns [11, 12].

We therefore sought to propose a hypnotic approach that would be able to relieve the burning sensations felt by patients receiving treatment with a capsaicin patch in the framework of follow-up for chronic neuropathic pain in relation to active cancer or its treatment (post-operative pain, chemotherapy-related toxicity). The aim of the study was to evaluate the impact of playing a standardized hypnosis recording during application of an 8% capsaicin patch, firstly on the pain induced by capsaicin treatment, and secondly, on the anxiety felt during application of the patch.

## Methods

### Study design and oversight

**Fig. 1:**
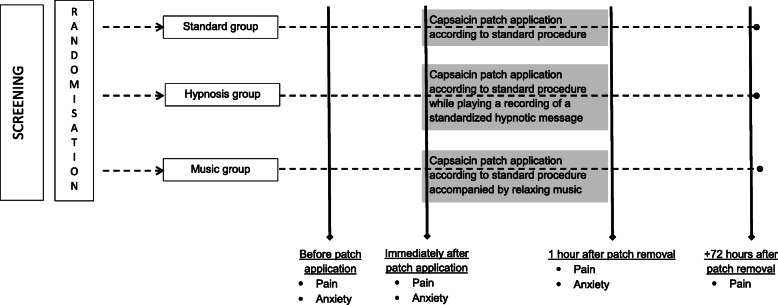
Flowchart of the study design

We performed a randomized, single-centre, prospective pilot study in three parallel groups at the Institut de Cancérologie de Lorraine (ICL, Nancy, France). Participants were randomly assigned between the following groups in a 1:1:1 ratio: (1) application of the capsaicin patch according to standard procedure, which includes the possibility for the patient to apply a cold patch on the affected zone (Standard group); (2) application of the capsaicin patch according to the standard procedure, while at the same time playing a recording of a standardized hypnotic message (Hypnosis group); and (3) application of the capsaicin patch according to the standard procedure, accompanied by relaxing music (Music group). Randomization was centralized by computer-generated random numbers in blocks of six. The study was not blinded owing to the practical barriers to masking. The study design is illustrated in Fig. 1. This study adhered to the principles of the Declaration of Helsinki and guidelines of Good Clinical Practice. The safety and ethical conformity of the study was reviewed and approved by the Ethics Committee (Comité de Protection des Personnes, CPP Est III) on 03/05/2016 under the number 16.03.06 and by the National agency for the safety of medicines and health products on 22/04/2016 under the number 160228B-32. The study was also registered with the ClinicalTrials.gov registry on 04/07/2016 under the number NCT02822625. All participants provided written informed consent.

### Inclusion and exclusion criteria

All patients were attending the Interdisciplinary Unit for Supportive Care for Oncology Patients at ICL. Patients were eligible if they had neuropathic pain, as diagnosed with the Douleur Neuropathique 4 Questions (DN4) questionnaire, and had previously had at least one application of a capsaicin patch, and had a new indication for capsaicin patch application. We excluded patients with psychotic disorders, patients with hearing disorders, patients unable to understand French, patients < 18 years old, persons under judicial protection or legal guardianship, patients requiring premedication with painkillers before capsaicin patch application, and patients with a history of hypersensitivity to capsaicin or to any of the patch excipients.

### Interventions

Patient screening was performed by the lead nurse from the pain management clinic (ML). After obtaining written informed consent, inclusion was performed on the day of the patch application in the outpatient unit. Then, patient randomization was performed. Study procedures were carried out in the same conditions for all patients, namely in the room located furthest away from the workstation in the unit, in order to minimize background noise, and to facilitate patient comfort and high-quality listening. In addition, a “Do not disturb” sign was placed on the door of the room to deter anyone from disturbing the patient during the time the patch was in place. The duration of capsaicin patch application was one hour. After patch removal, the patient was free to go home. A study nurse called the patient by phone at 72 h after the patch application for the final evaluation of pain.

The hypnotic message was created and recorded by a nurse hypnotist (RE) at our institution who is qualified to train other hypnosis practitioners. The full script was read and amended by a renowned scientific expert in the field of medical hypnosis (AB). The text of the script included the different elements that characterize a hypnosis session, taking account of the rhythm of suggestions and the tone of voice, adapted to the progress of the study procedures and accommodating the constraints related to the monitoring of the patient. The therapeutic metaphor used in the recording was a walk in the mountains, with a dip in a lake of cool water. The story was aligned with the different stages of application of the capsaicin patch (blood pressure measurement, inclusion of outside sounds, duration of application). Numerous hypnosis techniques are proposed in the script in addition to the metaphors (confusion, acceptance, double-binds…). The hypnotic message was recorded in a professional recording studio in order to minimize background noise and optimize the quality.

The relaxing music was chosen by the study investigators based on the advice of a music therapist who works with patients suffering from pain. Three musical atmospheres were chosen (classical, easy-listening and relaxing), in order to allow the patients to choose whichever type of music best corresponded to their expectations and personal tastes.

All recordings (hypnotic message and musical excerpts) were transferred to an MP3 player to make listening easier for the patient and played through a high-definition audio headset (NEW APPLE iPod Nano 16Go Space, APPLE®).

### Outcome measures

Assessments were conducted at 4 different times (see Fig. 1). Data recorded for the study purposes was entered into an electronic Case Report Form (CRF) (Cleanweb®, Télémédecine SA Boulogne-Billancourt, France). We recorded gender, age, history of cardiovascular disease, concomitant treatments, pain and anxiety evaluations, patient’s perception of the time the patch was in place, application of cold pack while the patch was in place. For patients randomized to the Hypnosis group, we also noted whether the patient listened to the hypnotic message in its entirety (yes/no). For patients randomized to the Music group, we noted the type of music the patient chose (classical, easy-listening, relaxing). The primary outcome was pain measured using a numerical rating scale (NRS). NRS is a one-dimensional measurement of pain intensity in adults. The NRS form was filled out by reporting scores verbally. The NRS score ranges from 0 to 10, with 0 indicating no pain and 10 the worst imaginable pain. Higher scores on the NRS scale indicate a higher intensity of pain [13]. Secondary outcomes were anxiety measured using a NRS [14], with 0 indicating no anxiety and 10 the worst imaginable anxiety, and patient’s perception of the length of time the patch was in place (in minutes).

### Statistical methods

Quantitative variables are described as mean ± standard deviation (SD) if normally distributed, or median [interquartiles] if non-normally distributed, and were compared using ANOVA or the Kruskal-Wallis test. The normality of the distribution was investigated by the Shapiro-Wilks test. Qualitative variables are described as number (percentage) and were compared using the Chi square or Fisher’s exact test as appropriate.

The primary endpoint was pain as assessed by the NRS (from 0, corresponding to no pain, to 10, intolerable pain) at the time of patch removal. Comparisons of pain and anxiety immediately after patch application and removal were performed by analysis of covariance (ANCOVA) in order to adjust for values prior to application. In case of differences across the three groups, Bonferroni correction was applied to account for multiple comparisons.

All analyses were performed using SAS version 9.4 (SAS Institute Inc., Cary, NC). All tests were two-sided and a *p*-value < 0.05 was considered statistically significant.

The average pain score after application of a capsaicin patch is estimated at 6 ± 2 [7]. A difference of at least 2 points is considered clinically meaningful. Based on the hypothesis of an average pain score of 5 in the hypnosis group, and 7 in the control group, 23 patients were required in each group to show a significant difference with power of 80% and an alpha risk of 1.6% (after Bonferroni correction to account for the three groups), i.e. 69 patients in total.

## Results

### Patients

**Fig. 2:**
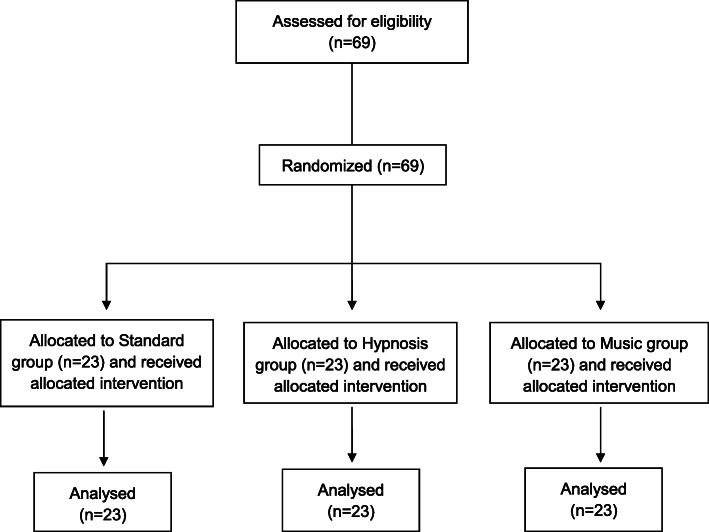
Flow Diagram

From August 2016 to July 2019, a total of 69 patients were included; 23 were randomized to each arm (Fig. 2). Fourteen participants were men (20%), 55 (80%) were women. The baseline characteristics of the study population are shown in Table 1. There was no significant difference between groups in terms of age, history of cardiovascular disease, presence of signs or symptoms at inclusion or ongoing treatment. There was also no significant difference between groups in the level of pain or anxiety before the patch was applied (Table 1).

**Table 1:** Baseline characteristics of the study population according to randomization group

	TOTAL ***N*** = 69	STANDARD ***N*** = 23	HYPNOSIS ***N*** = 23	MUSIC ***N*** = 23	***p***-value
Gender, n(%)					0.534
Males	14 (20.3)	6 (26.1)	3 (13)	5 (21.7)	
Females	55 (79.7)	17 (73.9)	20 (87)	18 (78.3)	
Age (years), mean ± SD	57.4 ± 12.8	58.7 ± 13	53.3 ± 14.9	58.7 ± 10	0.265
History of cardiovascular disease	25 (36.2)	11 (47.8)	7 (30.4)	7 (30.4)	0.366
Ongoing cardiovascular treatment at inclusion ^a^	22/25 (88.0)	11/11 (100)	5/7 (71.4)	6/7 (85.7)	–
Level II or III analgesic	26 (37.7)	10 (43.5)	8 (34.8)	8 (34.8)	0.781
Antidepressants or antiepileptics for neuropathic pain	33 (47.8)	10 (43.5)	12 (52.2)	11 (47.8)	0.840
Benzodiazepine or antidepressants for depression/anxiety disorders	14 (20.3)	6 (26.1)	3 (13.0)	5 (21.7)	0.534
Previously had hypnosis	19 (27.5)	4 (17.4)	9 (39.1)	6 (26.1)	0.504
Number of prior hypnosis sessions, median [IQR] ^b^	2.5 [1;5]	2.5 [1;9.5]	3 [2;5]	2 [1;4]	–
Pain score prior to patch application^c^, mean ± SD	3.7 ± 2.4	4.5 ± 2.3	3.2 ± 2.6	3.4 ± 2.2	0.196
Anxiety score prior to patch application^c^, mean ± SD	2.6 ± 2.9	3.9 ± 3.1	2.3 ± 2.7	1.9 ± 2.7	0.096

*Abbreviations*: *SD* standard deviation, *IQR* interquartile range

^a^ Among the 25 patients with a history of cardiovascular disease

^b^ Among the 19 patients who previously had hypnosis

^c^ Numerical rating scale (NRS). The NRS score ranges from 0 to 10, with 0 indicating no pain/anxiety and 10 the worst imaginable pain/anxiety. Higher scores on the NRS scale indicate a higher intensity of pain/anxiety

In the music group, 14 patients (60.9%) chose easy-listening music, 5 (21.7%) chose classical music, and 3 (13%) chose relaxing music. Twenty patients (90.9%) listened the hypnotic message in its entirety. A cold pack was applied by 18 patients (78.3%) in the standard group, 17 (73.9%) in the hypnotic group and, 20 (87.0%) in the music group (*p* = 0.652).

### Primary outcome

Overall, there was no significant difference in pain scores between groups after application of the capsaicin patch (*p* = 0.355) (Table 2).

**Table 2:** Pain and anxiety before and after application of a capsaicin patch and duration of patch application as perceived by the patient in the three study arms

	STANDARD ***N*** = 23	HYPNOSIS ***N*** = 23	MUSIC ***N*** = 23	***p***-value
	Mean ± SD	Mean ± SD	Mean ± SD	
Pain ^a^				
Before application	4.5 ± 2.3	3.2 ± 2.6	3.4 ± 2.2	0.196
Immediately after application	4 ± 2.2	3.0 ± 2.4	3.8 ± 2	0.245 *
After patch removal	6.4 ± 2.9	4.6 ± 2.9	5.3 ± 2.6	0.355 *
72 h after patch removal	3.4+/−2.9	3.2+/− 2.9	2.1+/− 2.7	0.461 *
Anxiety ^a^				
Before application	3.9 ± 3.1	2.3 ± 2.7	1.9 ± 2.7	0.100
Immediately after application	4.0 ± 3.2	2.3 ± 2.7	1.9 ± 2.7	0.783 *
After patch removal	4.2 ± 3.2	1.2 ± 2.5	1.7 ± 2.5	0.010 *
Duration of patch application as perceived by the patient, minutes	63.8 ± 10.7	44.6 ± 16.5	49.3 ± 14.7	0.002

*Abbreviations*: *SD* standard deviation

^a^ Numerical rating scale (NRS). The NRS score ranges from 0 to 10, with 0 indicating no pain/anxiety and 10 the worst imaginable pain/anxiety. Higher scores on the NRS scale indicate a higher intensity of pain/anxiety

* Adjusted for values prior to patch application

### Secondary outcomes

For anxiety (Table 2), scores were significantly different between groups after patch removal (*p* = 0.010). Compared to standard application, anxiety was significantly lower in the hypnosis group after the application (*p* = 0.007 after Bonferroni adjustment). There was no significant difference between the standard and music arms, or between the hypnosis and music arms (*p* = 0.271 and *p* = 0.423 respectively after Bonferroni adjustment). There was also a significant difference between groups in terms of the perceived duration of the application (*p* = 0.002, Table 2), with a significant difference between the standard and hypnosis arms (*p* < 0.001 after Bonferroni adjustment), and between the standard and music arms (*p* < 0.001 after Bonferroni adjustment), but not between the hypnosis and music arms (*p* = 0.424 after Bonferroni adjustment).

## Discussion

This study aimed to address the adverse effects that may occur during application of a capsaicin patch (e.g. burning or tingling sensation, intensification of pain) by proposing a standardized hypnotic message to be played while the patch was in place. Even if expected results on pain reduction were not obtained, patients who listened to the hypnosis recording had a significantly lower level of anxiety compared to the standard patch application procedure. We included a group that listened to music during the application of the patch since the effects of music on pain and anxiety have previously been demonstrated [15] and could serve as a reference to explain the psychological and neurophysiological mechanisms at play in this process. Our study precludes any conclusions regarding the efficacy of music on anxiety. To the best of our knowledge, our study is the first to assess the impact of a standardized hypnotic message on self-reported pain and anxiety. What is innovative in this approach is that the hypnosis session offered to patients was recorded. Indeed, in normal clinical practice, the sessions are “tailor-made”, individualized for each patient during a therapeutic encounter. Here, to a certain extent, we can say that the technical dimension of hypnosis was evaluated, without the individualized relationship, since the session was standardized. The aim was to evaluate a practice from which patients could benefit in all cases, even when the caregiver is not trained to accompany them with hypnosis.

We did not observe any difference in pain scores between groups, which is surprising in view of the results previously obtained with hypnosis for pain [16]. Perhaps the personalized approach to hypnosis, which more closely corresponds to the reality of hypnosis practices in real life, might have yielded a more marked difference. Additionally, the metaphor used in the message was that of going walking in the mountains and bathing in a mountain lake. It would also be interesting to assess the effect of using specific analgesic suggestions, such as the glove anesthesia method, or modulation of pain through mental imagery. This strategy differs from the simple suggestion of coolness, and might help patients to experience less pain.

We observed that pain and anxiety scores tended to be lower before patch application in patients in the hypnosis and music arms, as compared to those in the standard treatment arm. This observation could be explained by a possible placebo effect when the patient is informed of the treatment group they have been assigned to by the randomization process [17, 18]. Indeed, the patient was informed of the allocated treatment group before the first evaluation of pain and anxiety. A previous study has shown that labelling the induction process as “hypnosis” led to a significant increase in patient suggestibility, compared to when it was just called “relaxation” [19]. The patients’ expectations of the therapeutic accompaniment appear to be a key factor in their subjective experience of a potentially painful and anxiogenic care process.

It is noteworthy that patients in the hypnosis and music arms had a subjective impression that the patch was in place for a much shorter time compared to patients in the standard treatment arm. This distorted perception of time can be explained by several factors. Firstly, by the simple fact of distracting attention by listening to, and concentrating on the audio recording. Secondly, temporal distortion has been described as a characteristic of the hypnotic phenomenon [20, 21]. We believe that this finding of temporal foreshortening is important, as it contributes to limiting the discomfort caused by exposure to the patch, in comparison with patients who are treated in the standard manner, without accompaniment.

### Study limitations

It should be underlined that all patients included in this study had previously had a capsaicin patch applied, and were attending for a second application. Although the purpose of this was to ensure a homogeneous population, it is possible that the patients were already familiar with the sensations, and could anticipate them once informed of their randomization group. Secondly, although the one-item question used in this study is a validated method to assess anxiety [14], we did not use a common instrument such as the State form of the Spielberger State Trait Anxiety Inventory (STAI). However, the STAI comprises 20 items, and in our study, anxiety was measured at several timepoints over a short period of time. Therefore, the STAI did not seem to be the most suitable tool in this context, and we opted instead for a numerical rating scale. Finally, our study failed to show any superiority of hypnosis over music, probably due to a lack of statistical power, since the study was designed to show a difference between the hypnosis group and the standard procedure.

### Clinical implications

Patients’ expectations of analgesic therapy, maintaining an acceptable level of anxiety, and the perception of the passage of time during the delivery of healthcare, all appear to be determinant in shaping the patient’s overall subjective experience. Our study focused on cancer patients with at least one previous application of a capsaicin patch. Since the standardized hypnosis message is easy to use, without the need for additional human resources, its use could easily be generalized to all patients receiving treatment with a capsaicin patch. It would be also interesting to investigate the same procedure among patients who were having a patch applied for the first time and who had never previously been exposed to capsaicin.

## Conclusions

In this study, although it had no effect on the perceived level of pain as compared to standard procedures, listening to a standardized hypnotic message during application of a capsaicin patch was found to lower anxiety. This study opens interesting avenues for improving the comfort of patients during treatment. These findings warrant further evaluation in other indications where treatments may generate pain or anxiety using specific analgesic suggestions rather than suggestions only based on sensations. Using recorded standardized hypnotic messages and procedures could enable the use of this approach in healthcare centers with limited facilities.

## Data Availability

The datasets used and/or analysed during the current study are available from the corresponding author on reasonable request.
